# Retraction: Bak Compensated for Bax in p53-null Cells to Release Cytochrome c for the Initiation of Mitochondrial Signaling during Withanolide D-Induced Apoptosis

**DOI:** 10.1371/journal.pone.0228839

**Published:** 2020-02-03

**Authors:** The PLOS ONE Editors

After this article [1] was published, concerns were raised about results reported in Figs 1, 2, and 6.

Specifically:

- The same β-actin data are reported for each cell line in Fig 1C and 1D, and similarly the same β-actin data are reported for each cell line in Fig 2C and 2D. The authors explained that panels C and D of each of these figures report results of experiments using the same lysates from the same experiments. Equal amounts of the same protein samples were run on parallel gels to obtain the data reported in panels C and D, as such, the same β-actin controls applied.
- In Fig 6D, for Spleen data, it appears as though the right half of the 40X untreated panel overlaps with the left half of the 40X WithaD panel. The authors noted that this was due to an error in preparing the WithaD panel and provided a replacement image.
- In Fig 6D, there are several regions which appear to be repeated within the 20X Spleen Untreated panel, and between the 20X Spleen Untreated and 20X Spleen WithaD panels. The authors commented that animal experiments and subsequent histology were conducted at two different institutes and were unable to clarify the reason(s) for or origins of the similarities. The authors offered replacement data which they noted were obtained by staining additional sections from the original paraffin embedded tissues for this experiment.

In follow-up discussions, the authors provided body weight and tumor weight data to support results statements referencing “data not shown”, and they noted that all data supporting the results in the article are available except for the original flow cytometry output files.

The unresolved concerns about the 20X Spleen data reported in Fig 6D call into question the integrity of the results reported in this figure. In light of this issue, the *PLOS ONE* Editors retract this article.

CM notified the journal that all authors disagree with the retraction. SM and KB confirmed their disagreement. The other authors either could not be reached or did not reply directly.
